# Systolic Versus Diastolic Echocardiographic Assessment of Epicardial Adipose Tissue for the Detection of Obstructive Coronary Artery Disease: A Systematic Review and Meta-Analysis

**DOI:** 10.3390/jcm15020878

**Published:** 2026-01-21

**Authors:** Andrea Sonaglioni, Giulio Francesco Gramaglia, Gian Luigi Nicolosi, Massimo Baravelli, Michele Lombardo

**Affiliations:** 1 Division of Cardiology, IRCCS MultiMedica, 20123 Milan, Italy; massimo.baravelli@multimedica.it (M.B.); michele.lombardo@multimedica.it (M.L.); 2 Department of Emergency, Fondazione IRCSS Ca’ Granda, Ospedale Maggiore Policlinico, 20122 Milan, Italy; giulio.gramaglia@unimi.it; 3 Division of Cardiology, Policlinico San Giorgio, 33170 Pordenone, Italy; gianluigi.nicolosi@gmail.com

**Keywords:** epicardial adipose tissue, coronary artery disease, echocardiography, obstructive coronary stenosis, meta-analysis

## Abstract

**Background**: Epicardial adipose tissue (EAT) is a metabolically active visceral fat depot increasingly associated with the development and progression of coronary artery disease (CAD). Transthoracic echocardiography is the most widely used modality for EAT assessment; however, substantial heterogeneity exists regarding the timing of measurement within the cardiac cycle, with EAT thickness variably assessed during systole or diastole. Whether these measurements provide equivalent information for identifying obstructive CAD remains unclear. This systematic review and meta-analysis evaluated the association between echocardiographically measured EAT thickness and angiographically confirmed obstructive CAD, with specific focus on systolic versus diastolic assessments. **Methods**: PubMed, Scopus, and EMBASE were systematically searched through December 2025 for observational studies comparing EAT thickness in patients with and without obstructive CAD confirmed by invasive coronary angiography. Random-effects models were used to pool standardized mean differences (SMDs) for systolic and diastolic EAT thickness. Heterogeneity was assessed using the I^2^ statistic, publication bias by funnel plots and Egger’s regression test, and robustness by meta-regression and leave-one-out sensitivity analyses. **Results**: Twenty-two studies including more than 6500 patients were analyzed. Both systolic and diastolic EAT thickness were significantly greater in patients with obstructive CAD than in non-CAD controls. Systolic EAT showed a large, pooled effect size (SMD 1.27; 95% CI 0.96–1.59; *p* < 0.001), while diastolic EAT demonstrated a similarly strong association (SMD 1.59; 95% CI 1.10–2.07; *p* < 0.001). Heterogeneity was substantial (I^2^ > 90%), but the direction of effect was consistent across all studies. Meta-regression analyses indicated that demographic, clinical, metabolic, geographic, and methodological characteristics, including ultrasound software/vendor category and timing of EAT measurement, did not significantly moderate the association between EAT thickness and obstructive CAD. No significant publication bias was detected, and sensitivity analyses confirmed the robustness of the results. **Conclusions**: Echocardiographically measured EAT thickness is strongly and consistently associated with obstructive CAD, irrespective of whether measurements are obtained during systole or diastole. Although both approaches show robust discriminatory capacity at the population level, differences in effect magnitude suggest that they may not be fully interchangeable. Moreover, in the absence of standardized and broadly applicable cut-off values, the interpretation and clinical management of EAT measurements as individual risk predictors require further investigation.

## 1. Introduction

Obstructive coronary artery disease (CAD) remains a leading cause of morbidity and mortality worldwide, despite substantial advances in preventive strategies, diagnostic imaging, and revascularization techniques [1]. Early identification of individuals at increased risk of obstructive CAD is therefore of paramount importance, particularly in clinical contexts where traditional cardiovascular risk factors alone may not fully explain individual susceptibility to coronary atherosclerosis [2].

Epicardial adipose tissue (EAT) has gained increasing attention as a metabolically active visceral fat depot located between the myocardium and the visceral layer of the pericardium, surrounding the epicardial coronary arteries [3]. Unlike other adipose compartments, EAT shares a common microcirculation with the underlying myocardium and lacks a separating fascial plane, allowing direct paracrine and vasocrine interactions with the coronary vasculature [4]. Through the secretion of pro-inflammatory cytokines, adipokines, and free fatty acids, EAT has been associated with endothelial dysfunction, plaque formation, and progression of coronary atherosclerosis [5]. Accordingly, increased EAT thickness has been consistently correlated with both the presence and angiographic severity of CAD across multiple imaging modalities [6,7].

Transthoracic echocardiography (TTE) represents the most widely available, cost-effective, and clinically applicable technique for EAT assessment [8]. Nevertheless, despite its broad adoption, considerable heterogeneity persists in echocardiographic EAT quantification, particularly regarding the timing of measurement within the cardiac cycle. Some investigators measure EAT thickness at end-systole, when epicardial fat is maximally compressed against the myocardium, whereas others perform measurements at end-diastole, when ventricular relaxation alters the spatial relationship between EAT, myocardium, and pericardium [9,10]. These approaches are often considered interchangeable, although they reflect different mechanical and anatomical conditions.

Importantly, the behavior of EAT is not static throughout the cardiac cycle. Cyclic changes in ventricular volume, myocardial wall stress, and pericardial constraint may influence the apparent thickness of EAT, with potential implications for measurement reproducibility and its association with coronary pathology [11]. Whether systolic and diastolic EAT measurements convey equivalent information regarding obstructive CAD risk therefore remains uncertain. Although individual studies have demonstrated significant associations between EAT thickness and CAD using either systolic or diastolic measurements, no consensus has emerged as to which phase of the cardiac cycle provides superior discriminatory or predictive value.

Previous meta-analyses have primarily examined the relationship between EAT and CAD without accounting for the timing of echocardiographic assessment, implicitly assuming equivalence between systolic and diastolic measurements [12,13,14]. This assumption may partly explain the substantial heterogeneity reported across studies and limits the clinical interpretability of pooled estimates. A systematic comparison of systolic versus diastolic EAT measurements is therefore needed to determine whether these approaches capture the same biological signal or reflect distinct aspects of epicardial fat–coronary interactions.

Accordingly, the present systematic review and meta-analysis aimed to evaluate the association between echocardiographically measured EAT thickness and angiographically confirmed obstructive CAD, with a specific focus on comparing measurements obtained during systole and diastole. By conducting separate quantitative syntheses based on the timing of EAT assessment within the cardiac cycle, we sought to clarify whether systolic and diastolic EAT measurements differ in their ability to discriminate patients with obstructive CAD from those without significant coronary stenosis. We hypothesized that while both measurements would be significantly associated with obstructive CAD, the strength of this association would vary according to the phase of the cardiac cycle in which EAT is measured.

## 2. Materials and Methods

This systematic review and meta-analysis was conducted in accordance with the Preferred Reporting Items for Systematic Reviews and Meta-Analyses (PRISMA) guidelines [15] (Supplementary Materials S1).

The review protocol was prospectively registered in the INPLASY database (registration ID: INPLASY202610009) on 3 January 2026 (Supplementary Materials S2), prior to data extraction and statistical analysis.

### 2.1. Search Strategy

A comprehensive literature search was independently performed by two investigators (A.S. and G.G.) to identify all relevant studies evaluating EAT thickness in patients with and without obstructive CAD. PubMed, Scopus, and EMBASE databases were systematically searched from inception to December 2025.

The search strategy combined controlled vocabulary and free-text terms related to EAT, CAD, and TTE. The following keywords and Boolean operators were used: “epicardial adipose tissue” OR “epicardial fat” OR “epicardial fat thickness” AND “coronary artery disease” OR “obstructive coronary artery disease” OR “coronary stenosis” OR “coronary angiography” AND “echocardiography” OR “transthoracic echocardiography.”

No restrictions were applied regarding language, publication date, or study design. To ensure completeness, the reference lists of all eligible articles and relevant review papers were manually screened for additional studies not captured by the initial database search.

Any disagreements between reviewers during the study selection process were resolved through consensus. When consensus could not be reached, a third investigator was consulted to adjudicate eligibility.

### 2.2. Eligibility Criteria

Studies were considered eligible for inclusion if they met all of the following criteria: (1) observational study design (case-control or cross-sectional) directly comparing patients with angiographically confirmed obstructive CAD and individuals without significant coronary stenosis; (2) quantitative assessment of EAT thickness performed by TTE; (3) diagnosis of obstructive CAD established by invasive coronary angiography, using predefined thresholds of luminal stenosis in at least one major epicardial coronary artery; and (4) availability of extractable quantitative data on EAT thickness for both CAD and non-CAD groups, reported either as mean ± standard deviation or in a format allowing data transformation.

Studies were excluded if they met any of the following criteria: (1) assessment of EAT using imaging modalities other than TTE, such as cardiac computed tomography or cardiac magnetic resonance imaging, without echocardiographic measurements; (2) inclusion of heterogeneous populations without a clearly defined comparison between obstructive CAD and non-obstructive or normal coronary arteries; (3) absence of invasive coronary angiography as the reference standard for CAD diagnosis; (4) lack of quantitative EAT measurements or insufficient data to calculate effect estimates; and (5) non-original publications, including conference abstracts, editorials, letters, case reports, or narrative reviews.

### 2.3. Study Selection and Data Collection

Two reviewers (A.S. and G.G.) independently screened all retrieved records by title and abstract, followed by a full-text evaluation of potentially eligible articles. Study selection was performed according to the predefined eligibility criteria, and any disagreement was resolved through discussion and consensus.

Data extraction was carried out independently by the same reviewers using a standardized and predefined data collection form. Extracted information included (1) study characteristics, such as first author, year of publication, country, study design, and sample size of the CAD and non-CAD groups; (2) demographic and anthropometric variables, including age, sex distribution, body mass index (BMI), waist circumference, and waist-to-hip ratio, when available; (3) cardiovascular risk factors, namely hypertension, diabetes mellitus, dyslipidemia, active smoking, and family history of CAD; (4) laboratory parameters, including hemoglobin, serum creatinine, fasting plasma glucose, lipid profile components (low-density lipoprotein cholesterol, high-density lipoprotein cholesterol, and triglycerides), uric acid, and high-sensitivity C-reactive protein; (5) echocardiographic parameters, comprising left ventricular ejection fraction (LVEF), left ventricular mass, and EAT thickness assessed by TTE, with systolic and diastolic EAT measurements extracted separately when reported, together with any study-specific EAT cut-off values associated with obstructive or severe CAD; (6) clinical and angiographic indices, such as Gensini [16] and SYNTAX [17] scores, reflecting coronary atherosclerotic burden and lesion complexity; (7) baseline medical therapy, including use of antiplatelet agents, beta-blockers, calcium channel blockers, angiotensin-converting enzyme inhibitors, angiotensin receptor blockers, diuretics, nitrates, and statins; and (8) summary statistics, including mean ± standard deviation or median with interquartile range, along with corresponding *p*-values or confidence intervals.

When numerical results were reported exclusively in graphical form, data were extracted using WebPlotDigitizer (version 4.6), a validated digital measurement tool. When required for quantitative synthesis, medians and interquartile ranges were converted to means and standard deviations using established statistical methods. All extracted data were cross-checked for accuracy, and discrepancies between reviewers were resolved by re-examination of the original articles until consensus was achieved.

### 2.4. Assessment of Methodological Quality and Bias

The methodological quality and risk of bias of the included studies were independently assessed by two investigators (A.S. and G.L.N.) using the National Institutes of Health (NIH) Quality Assessment Tool for Case–Control Studies [18]. This instrument evaluates multiple domains related to study design, population selection, exposure assessment, and control of confounding.

Based on the number of criteria fulfilled, each study was assigned an overall quality rating of “good,” “fair,” or “poor” in accordance with NIH recommendations. Inter-rater agreement between reviewers was quantified using Cohen’s kappa coefficient (κ). Any disagreements in individual domain judgments or overall quality classification were resolved through discussion until consensus was reached.

### 2.5. Statistical Analysis

For descriptive purposes, continuous variables were summarized as medians with interquartile ranges (IQRs), while categorical variables were reported as counts and percentages. Comparative meta-analyses were performed using random-effects models. For continuous variables, pooled estimates were expressed as standardized mean differences (SMDs), whereas odds ratios were calculated for categorical variables when applicable. Statistical significance of pooled effects was assessed using Z-tests.

Because individual patient data were not available, formal assessment of data normality across studies using tests such as the Shapiro–Wilk test was not feasible. Instead, distributional characteristics were inferred based on the type of summary statistics reported in the primary studies. As most investigations presented continuous variables as medians with IQRs, the data were considered potentially non-normally distributed. Accordingly, descriptive statistics were retained in their original non-parametric form, and no transformation to means and standard deviations was applied for descriptive reporting.

For quantitative synthesis, echocardiographic parameters—including EAT thickness measured in systole and diastole, as well as LVEF—were pooled using SMDs. When required, means and standard deviations were estimated from medians and interquartile ranges using validated methods [19,20]. These conversions rely on parametric assumptions and therefore introduce additional uncertainty, particularly when underlying distributions deviate from normality. For this reason, converted values were used exclusively for meta-analytic pooling and not for descriptive summaries.

Separate meta-analyses were conducted according to the timing of EAT measurement within the cardiac cycle (systolic versus diastolic assessment). A random-effects model based on the DerSimonian–Laird method was selected a priori to account for anticipated clinical and methodological heterogeneity among studies. Between-study heterogeneity was quantified using the I^2^ statistic, with values of approximately 25%, 50%, and 75% indicating low, moderate, and high heterogeneity, respectively.

Publication bias was evaluated through visual inspection of funnel plots and formally tested using Egger’s regression asymmetry test. Meta-regression analyses were performed to explore potential sources of heterogeneity, including demographic variables (mean age and sex distribution), cardiovascular risk factors (prevalence of hypertension, diabetes mellitus, and active smoking), metabolic parameters (lipid profile components and waist circumference), geographic origin (Asian vs. non-Asian studies), and methodological characteristics related to echocardiographic assessment, including the timing of EAT measurement within the cardiac cycle (systolic vs. diastolic), ultrasound software/vendor category (GE-based platforms vs. non-GE systems), and the angiographic cut-off used to define obstructive CAD (≥50% luminal stenosis).

All statistical analyses were performed using Comprehensive Meta-Analysis software (version 3.0; Biostat, Englewood, NJ, USA) and IBM SPSS Statistics (version 29.0; IBM Corp., Armonk, NY, USA). All tests were two-tailed, and a *p*-value < 0.05 was considered statistically significant.

## 3. Results

### 3.1. Study Selection

**Figure 1:**
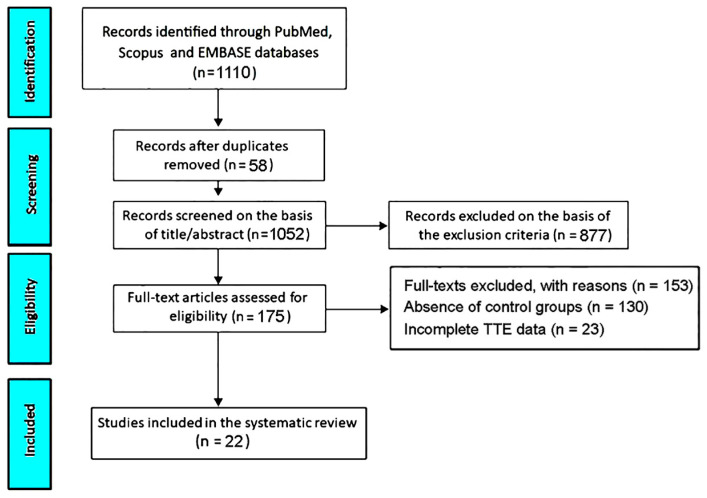
PRISMA flowchart depicting the study selection process. TTE, transthoracic echocardiography.

The initial search yielded 1110 records. Fifty-eight (5.2%) were removed as duplicates. A further 877 studies (79%) were excluded based on the predefined exclusion criteria. The remaining 175 studies (15.8%) were assessed for eligibility. Of these, 130 studies (11.7%) were excluded due to the absence of control groups, and 23 (2.1%) due to incomplete TTE data. Ultimately, 22 studies (2%) [21,22,23,24,25,26,27,28,29,30,31,32,33,34,35,36,37,38,39,40,41,42] were included in this systematic review and meta-analysis (Figure 1).

### 3.2. Clinical Findings

The 22 studies included in the present meta-analysis were published between 2007 and 2025 and were conducted across Asia, Europe, Africa, and the Americas. Specifically, studies originated from South Korea, Turkey, India, China, Japan, Iran, Egypt, Mexico, Cuba, Germany, Italy, Romania, and Tunisia. Overall, the included investigations enrolled a large cohort of patients undergoing invasive coronary angiography, comprising individuals with angiographically confirmed obstructive CAD and control subjects without significant coronary stenosis.

Most studies adopted a prospective, monocentric design, while only a minority were multicentric or retrospective. Across the selected literature, sample sizes varied widely, ranging from small exploratory cohorts to large observational populations exceeding several hundred participants. Patients with CAD were generally older and predominantly male compared with control groups, although demographic characteristics varied among studies.

EAT thickness was assessed in all studies using TTE. Measurements were most commonly performed on the free wall of the right ventricle from parasternal views, although the timing within the cardiac cycle differed across investigations. The majority of studies measured EAT at end-systole, defined according to ECG monitoring as the time point corresponding to the end of the T wave, whereas others adopted end-diastolic measurements identified at the peak of the R wave on the ECG, or reported values from both cardiac phases, thereby contributing to methodological heterogeneity across studies. Similarly, different echocardiographic platforms and software packages—including GE, Philips, Siemens, and Acuson systems—were used for EAT quantification.

All included studies defined obstructive CAD based on coronary angiography, most commonly using a threshold of ≥50% or ≥70% luminal stenosis in at least one major epicardial coronary artery. Several studies also explored EAT cut-off values associated with the presence of critical or severe CAD, although these thresholds showed substantial variability across populations, ranging approximately from 4.0 mm to over 10.0 mm. In some studies, no specific cut-off value was reported.

Overall, despite differences in study design, population characteristics, echocardiographic methodology, and EAT measurement protocols, all included studies provided comparative data on epicardial fat thickness between patients with obstructive CAD and those without significant CAD, forming the basis for the quantitative synthesis (Table 1).

### 3.3. Comparative Demographic and Clinical Characteristics of CAD and Non-CAD Cohorts

The demographic, clinical, laboratory, and echocardiographic characteristics of patients with obstructive CAD and those without significant coronary stenosis are summarized in Table 2.

Overall, data were derived from all 22 included studies, comprising more than 6500 patients undergoing invasive coronary angiography for suspected CAD.

From a demographic perspective, patients with obstructive CAD were significantly older than non-CAD controls (median age 62.1 vs. 56.6 years, *p* < 0.001) and showed a clear male predominance (66.4% vs. 51.1%, *p* < 0.001). Anthropometric indices revealed a more adverse visceral adiposity profile in the CAD group, with higher waist circumference (93.3 vs. 90.8 cm, *p* < 0.001) and waist-to-hip ratio (0.96 vs. 0.93, *p* = 0.002), whereas BMI did not differ significantly between groups (*p* = 0.27). These findings suggest that central fat distribution, rather than overall adiposity, was more closely associated with the presence of obstructive coronary disease across the included cohorts.

Traditional cardiovascular risk factors were markedly more prevalent among patients with CAD. Hypertension (63.1% vs. 47.5%, *p* < 0.001), diabetes mellitus (38.8% vs. 21.3%, *p* < 0.001), dyslipidemia (50.8% vs. 37.4%, *p* < 0.001), active smoking (41.6% vs. 29.3%, *p* < 0.001), and a family history of CAD (23.1% vs. 15.4%, *p* = 0.001) were all significantly more frequent in the CAD group, indicating a substantially higher cumulative cardiometabolic risk burden.

Laboratory parameters further reflected a more unfavorable metabolic and inflammatory profile in patients with CAD. Fasting plasma glucose levels were significantly higher (125.5 vs. 104.9 mg/dL, *p* = 0.001), while high-density lipoprotein cholesterol concentrations were lower (44.6 vs. 49.2 mg/dL, *p* < 0.001). Triglycerides (168.5 vs. 150.3 mg/dL, *p* < 0.001), serum uric acid (6.3 vs. 5.2 mg/dL, *p* = 0.002), and high-sensitivity C-reactive protein (1.31 vs. 0.81 mg/dL, *p* = 0.003) were also significantly elevated, consistent with greater metabolic dysregulation and systemic inflammation. Serum creatinine levels were modestly higher in the CAD group (*p* = 0.02), whereas hemoglobin levels were comparable between groups. Low-density lipoprotein cholesterol did not differ significantly (*p* = 0.42), likely reflecting heterogeneous lipid-lowering treatment strategies.

Echocardiographic assessment revealed relevant functional differences. LVEF was significantly lower in patients with CAD (52.8% vs. 58.4%, *p* < 0.001), although values were generally within a preserved or mildly reduced range. Left ventricular mass did not differ significantly (*p* = 0.19). In contrast, EAT thickness showed a striking and consistent increase in the CAD group, both when measured in systole (6.8 vs. 4.5 mm, *p* < 0.001) and in diastole (5.7 vs. 3.5 mm, *p* < 0.001). Importantly, measurement reproducibility (intraclass correlation coefficient, ICC) was reported only in a limited number of studies [33,35,42], but showed generally good agreement: for systolic EAT, intra-observer ICC ranged from 85.0% to 92.7% and inter-observer ICC from 83.0% to 94.3% (mean values 88.85% and 90.43%, respectively); for diastolic EAT, intra-observer ICC ranged from 78.0% to 96.0% and inter-observer ICC from 77.0% to 94.0% (mean values 87.00% and 88.00%, respectively). Reported EAT cut-off values associated with obstructive CAD clustered within a relatively narrow range across studies, underscoring the potential clinical relevance of EAT quantification at the population level.

Angiographic severity scores further differentiated the two cohorts. When available, both Gensini (44.6 vs. 10.9, *p* < 0.001) and SYNTAX scores (27.3 vs. 3.8, *p* < 0.001) were substantially higher in patients with CAD, confirming a greater atherosclerotic burden and lesion complexity.

Finally, baseline medical therapy differed between groups, with patients with CAD more frequently treated with antiplatelet agents, beta-blockers, calcium channel blockers, renin–angiotensin system inhibitors, nitrates, and statins (all *p* ≤ 0.04), reflecting both secondary prevention strategies and the higher prevalence of comorbid conditions.

Taken together, these findings delineate a distinct clinical phenotype associated with obstructive CAD, characterized by older age, male predominance, clustering of cardiovascular risk factors, adverse metabolic and inflammatory profiles, mildly reduced systolic function, and markedly increased epicardial fat thickness. These consistent differences provide the clinical framework for the subsequent quantitative analyses evaluating the association between EAT and obstructive CAD.

### 3.4. Meta-Analysis of the Predictive Value of Epicardial Adipose Tissue for Obstructive CAD

**Figure 2:**
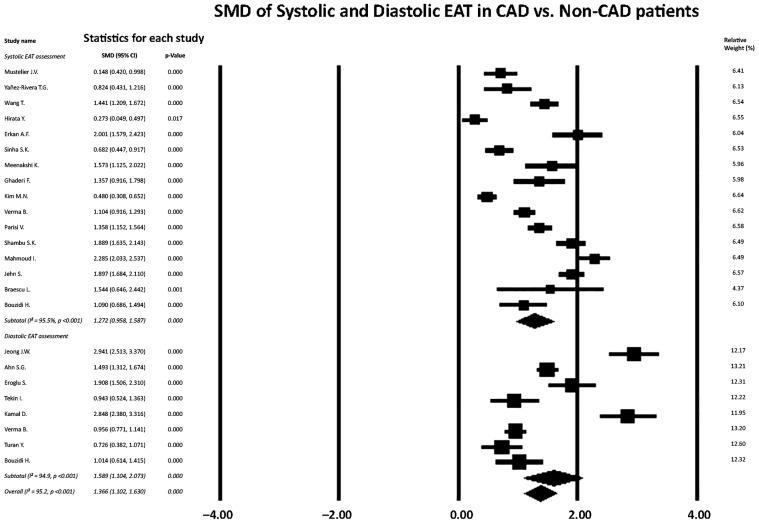
Forest plot of standardized mean differences in systolic [24,25,26,27,28,29,30,31,32,35,37,38,39,40,41,42] and diastolic [21,22,23,33,34,35,36,42] EAT thickness between CAD and non-CAD patients. Individual study estimates are represented by squares, with the size proportional to the study weight, and horizontal lines indicate 95% CIs. Diamonds represent pooled effect estimates for systolic EAT, diastolic EAT, and the overall analysis. A random-effects model was applied. Heterogeneity was assessed using the I^2^ statistic. Values to the right of zero indicate higher EAT thickness in CAD patients. CAD, coronary artery disease; EAT, epicardial adipose tissue.

The results of the meta-analysis evaluating the association between EAT thickness and the presence of obstructive CAD are summarized in Figure 2.

Separate quantitative analyses were performed according to the timing of EAT measurement within the cardiac cycle, distinguishing systolic from diastolic assessments.

Sixteen studies contributed to the meta-analysis of systolic EAT thickness. Pooled analysis demonstrated a significantly greater systolic EAT thickness in patients with obstructive CAD compared with non-CAD controls, with a large and statistically significant effect size (pooled SMD = 1.27, 95% CI 0.96–1.59, *p* < 0.001). Substantial heterogeneity was observed across studies (I^2^ = 95.5%, *p* < 0.001), reflecting variability in study populations, echocardiographic methodology, and measurement protocols; nevertheless, all included studies consistently showed higher systolic EAT values in the CAD group.

Eight studies were included in the meta-analysis of diastolic EAT thickness. Diastolic EAT was also significantly increased in patients with obstructive CAD compared with controls, with a pooled effect size indicating a strong association (SMD = 1.59, 95% CI 1.10–2.07, *p* < 0.001). As with systolic measurements, heterogeneity among studies was high (I^2^ = 94.9%, *p* < 0.001), but the direction of effect was uniform, with all studies reporting greater diastolic EAT thickness in the CAD cohort.

When both systolic and diastolic measurements were combined in an overall analysis, increased EAT thickness remained significantly associated with the presence of obstructive CAD (overall SMD = 1.37, 95% CI 1.10–1.63, *p* < 0.001; I^2^ = 95.2%, *p* < 0.001). This confirms a robust association between epicardial fat accumulation and angiographically proven CAD, irrespective of the timing of EAT assessment within the cardiac cycle.

Overall, these quantitative findings demonstrate that EAT measured by TTE is significantly associated with obstructive CAD. Both systolic and diastolic EAT measurements showed strong and consistent discriminatory capacity at the population level, supporting their use as imaging markers in the evaluation of patients undergoing coronary angiography.

**Figure 3:**
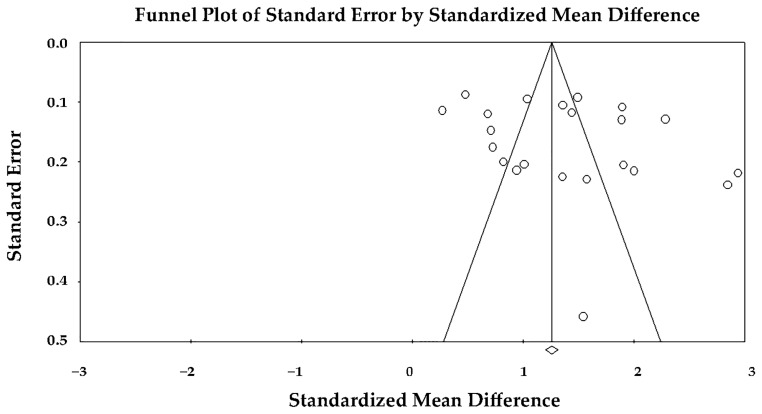
Funnel plot assessing publication bias for the association between epicardial adipose tissue thickness and coronary artery disease.

Despite the substantial heterogeneity observed across studies, the assessment of small-study effects did not suggest a relevant influence of publication bias. Visual inspection of the funnel plot showed an overall symmetric distribution of effect estimates around the pooled standardized mean difference (Figure 3), with no clear evidence of asymmetry across the range of standard errors.

This visual impression was supported by Egger’s regression test, which did not demonstrate a statistically significant deviation from symmetry (intercept = 4.05 ± 2.82; t(20) = 1.44; two-tailed *p* = 0.17). Accordingly, there was no formal evidence of small-study effects or publication bias influencing the pooled estimates. Therefore, the observed heterogeneity across studies was unlikely to be primarily driven by selective reporting or publication bias.

### 3.5. Meta-Regression and Sensitivity Analysis Results

Meta-regression analyses were conducted to investigate whether selected demographic, clinical, metabolic, methodological, and geographic study-level characteristics moderated the association between EAT thickness and obstructive CAD. The examined covariates included mean age, proportion of male participants, prevalence of hypertension, diabetes mellitus, and smoking, lipid profile parameters, waist circumference, geographic origin (Asian vs. non-Asian studies), timing of EAT measurement within the cardiac cycle (systolic vs. diastolic), ultrasound software/vendor category (non-GE vs. GE), and the angiographic cut-off used to define obstructive CAD.

None of the evaluated covariates demonstrated a statistically significant moderating effect on the association between EAT thickness and obstructive CAD. Demographic characteristics, cardiovascular risk factors, metabolic parameters, geographic origin, measurement phase, ultrasound software/vendor category, and angiographic definition of obstructive disease did not explain the observed between-study variability. These findings indicate that the association between increased EAT thickness and obstructive CAD remains consistent across different study populations, technical platforms, and methodological definitions, and suggest that residual heterogeneity is likely driven by unmeasured factors not captured at the aggregated study level (Table 3).

Sensitivity analyses were conducted using a leave-one-out approach to assess the robustness of the pooled estimates. Sequential exclusion of individual studies did not materially alter the overall results. Across all iterations, the direction of effect remained unchanged, consistently demonstrating higher EAT thickness in patients with obstructive CAD compared with non-CAD controls. The magnitude of the pooled effect size showed only modest variation, with SMDs ranging from 1.26 (95% CI 0.90–1.63; *p* < 0.001) to 2.20 (95% CI 0.78–3.62; *p* = 0.002), and statistical significance was preserved throughout all sensitivity analyses. These findings indicate that no single study exerted a disproportionate influence on the overall results, confirming the stability and robustness of the observed association between increased EAT thickness and obstructive CAD.

### 3.6. Publication Bias Assessment

Inter-rater agreement for the risk-of-bias evaluation was high, with Cohen’s kappa coefficient indicating substantial concordance between reviewers (κ = 0.80).

Based on the NIH Quality Assessment Tool for Case–Control Studies, the methodological quality of the included investigations was generally rated as fair to good, with the majority of studies meeting a large proportion of the predefined quality criteria (Supplementary Materials S3).

Across studies, research aims and case–control definitions were clearly specified, and echocardiographic protocols for EAT assessment were applied in a consistent and standardized manner. However, several recurring methodological limitations were identified. Formal sample size or power calculations were infrequently reported, and blinding of outcome assessors was often absent or insufficiently detailed. Limited adjustment for potential confounding variables was also observed in a subset of studies. While these issues may reduce internal validity, they are unlikely to have introduced a systematic distortion in the direction of the observed associations.

**Figure 4:**
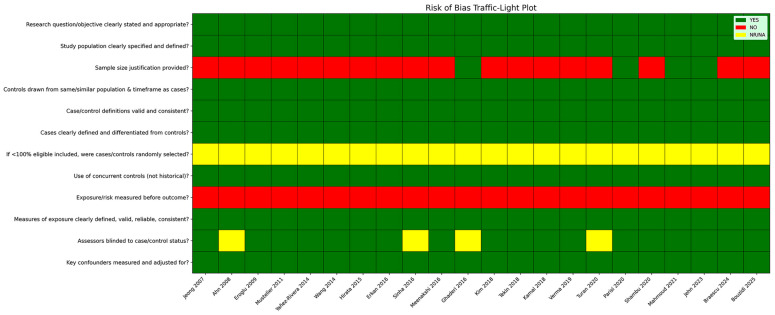
Traffic-light plot illustrating risk of bias among the included studies. The figure presents a traffic-light diagram summarizing the domain-level risk-of-bias evaluation for the 22 included studies [21,22,23,24,25,26,27,28,29,30,31,32,33,34,35,36,37,38,39,40,41,42], conducted according to the NIH Quality Assessment Tool for Case–Control Studies [18]. Color coding reflects the assessment for each methodological domain (green = “yes”, yellow = “not reported”, red = “no”).

To visually summarize the risk-of-bias assessment, a traffic-light plot displaying domain-specific judgments for each included study was generated (Figure 4).

**Figure 5:**
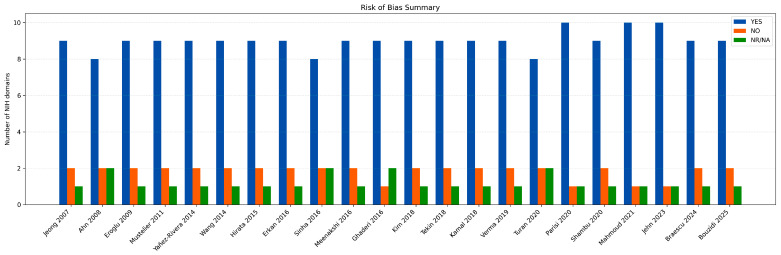
Risk of bias summary across included studies [21,22,23,24,25,26,27,28,29,30,31,32,33,34,35,36,37,38,39,40,41,42].

In addition, a summary bar chart illustrating the overall distribution of “YES”, “NO”, and “NR” ratings across the NIH assessment domains was constructed (Figure 5).

Overall, these visual representations emphasize consistent methodological gaps—most notably the lack of power calculations, incomplete blinding procedures, and residual confounding—which were common across the majority of the included studies.

## 4. Discussion

### 4.1. Synthesis of the Main Results

The present systematic review and meta-analysis provides the most comprehensive quantitative evaluation to date of the association between echocardiographically measured EAT thickness and angiographically confirmed obstructive CAD, with a specific focus on the timing of EAT assessment within the cardiac cycle. By separately analyzing systolic and diastolic EAT measurements, this study addresses an important methodological source of heterogeneity that has been largely overlooked in prior investigations and meta-analyses.

The main finding of this analysis is that increased EAT thickness is strongly and consistently associated with the presence of obstructive CAD at the population level, regardless of whether measurements are obtained during systole or diastole. Both systolic and diastolic EAT assessments demonstrated large and statistically significant pooled effect sizes, confirming epicardial fat accumulation as a robust imaging marker of coronary atherosclerotic burden. Importantly, although substantial between-study heterogeneity was observed, the direction of the association was uniform across all included studies, and the results remained stable in sensitivity analyses, supporting the reliability of the overall findings. Nevertheless, the consistently very high heterogeneity (I^2^ > 94% across analyses) indicates that pooled effect sizes should primarily be interpreted as population-level associations rather than precise estimates applicable to individual patients.

When examined separately, systolic and diastolic EAT measurements both showed strong discriminatory capacity for obstructive CAD. Diastolic EAT exhibited a numerically larger pooled effect size, whereas systolic EAT was supported by a greater number of contributing studies, reflecting its more frequent use in contemporary echocardiographic practice. These findings suggest that both phases capture clinically relevant information related to epicardial fat–coronary interactions, while also indicating that the magnitude of the association may vary according to the phase of the cardiac cycle in which EAT is measured.

Notably, meta-regression analyses did not identify demographic factors, cardiovascular risk profiles, metabolic parameters, geographic origin, ultrasound software/vendor category, timing of EAT measurement within the cardiac cycle, or the angiographic cut-off used to define obstructive CAD as significant moderators of the observed effect sizes. Specifically, study origin from Asian countries was not associated with significant differences in effect magnitude, indicating that ethnicity-related geographic variation did not materially contribute to the observed heterogeneity. This lack of explanatory power suggests that the association between increased EAT thickness and obstructive CAD is largely independent of these commonly reported study-level characteristics and remains consistent across different populations and methodological definitions. In this context, the wide range of proposed EAT cut-off values reported in the literature (approximately 4 mm to over 10 mm) is more likely driven by methodological heterogeneity—such as differences in imaging planes, measurement phase, caliper positioning, and acquisition protocols—rather than by ethnicity-specific biological differences alone, although population-related variability cannot be completely excluded.

From a methodological perspective, the persistence of high heterogeneity despite consistent effect direction and the absence of significant moderators in meta-regression highlights the likely contribution of unmeasured factors, including differences in echocardiographic acquisition and measurement protocols, anatomic landmarks used for EAT assessment, operator-dependent variability, and population-specific characteristics not captured in aggregated study-level data. Importantly, this degree of heterogeneity constrains the clinical interpretability of pooled estimates, particularly with regard to defining universal diagnostic thresholds or cut-off values for use in routine echocardiographic practice. Nevertheless, the absence of publication bias and the robustness of pooled estimates across leave-one-out analyses indicate that the observed association between EAT thickness and obstructive CAD is not driven by individual studies or selective reporting.

Taken together, the concordant results of sensitivity analyses and meta-regression support the stability of the observed association between increased EAT thickness and obstructive CAD, while also indicating that the substantial residual heterogeneity could not be attributed to the examined demographic, clinical, metabolic, geographic, or methodological covariates. This pattern suggests that variability across studies is likely influenced by additional unmeasured factors, including technical aspects of echocardiographic acquisition, center-specific measurement protocols, operator-related variability, and population characteristics not captured at the aggregated study level.

Collectively, these findings reinforce the concept that EAT is not merely a passive fat depot but a clinically meaningful marker of coronary atherosclerosis. At the same time, they underscore the importance of considering the timing of EAT measurement within the cardiac cycle when interpreting echocardiographic data. While both systolic and diastolic EAT measurements appear informative, their non-equivalence should be acknowledged, particularly in research settings and when defining diagnostic cut-off values. Given the very high between-study heterogeneity, EAT thickness should currently be viewed as a qualitative or semi-quantitative risk marker rather than a precise individual-level predictor. Overall, the present findings support the integration of EAT assessment into echocardiographic evaluation of patients with suspected CAD, while emphasizing the need for greater methodological standardization to optimize its clinical applicability. Indeed, owing to the lack of standardized and generalizable cut-off values, it remains to be clearly established how individual EAT measurements should be interpreted and managed as specific risk predictors at the level of the individual patient.

### 4.2. Pathophysiological Mechanisms Linking Epicardial Adipose Tissue and Obstructive Coronary Artery Disease

The strong and consistent association observed between increased EAT thickness and obstructive CAD in the present meta-analysis is supported by a growing body of experimental and clinical evidence implicating EAT as an active participant in coronary atherogenesis rather than a passive fat depot [43,44]. The unique anatomic and functional properties of EAT provide a plausible mechanistic framework through which local adiposity may directly influence coronary artery structure and function.

EAT is contiguous with the adventitia of the epicardial coronary arteries and shares the same microcirculation as the underlying myocardium, without an intervening fascial barrier. This intimate anatomical relationship facilitates direct paracrine and vasocrine signaling between EAT and the coronary vessel wall [45]. Under physiological conditions, EAT exerts cardioprotective effects by supplying free fatty acids for myocardial metabolism and secreting anti-inflammatory adipokines. However, in the setting of excess accumulation and metabolic dysregulation, EAT undergoes a phenotypic shift toward a pro-inflammatory, pro-atherogenic profile [46].

Hypertrophied epicardial adipocytes exhibit increased secretion of inflammatory cytokines such as interleukin-6, tumor necrosis factor-α, and monocyte chemoattractant protein-1, alongside reduced production of protective adipokines including adiponectin. This altered secretory milieu promotes endothelial dysfunction, oxidative stress, and vascular inflammation within adjacent coronary segments [47]. Histopathological and imaging studies have demonstrated that regions of increased EAT thickness frequently colocalize with more advanced coronary plaque burden, greater plaque vulnerability, and impaired endothelial-dependent vasodilation, supporting a local rather than systemic mechanism [48,49,50].

Beyond inflammation, EAT contributes to atherogenesis through multiple additional pathways. Increased lipolytic activity within epicardial fat leads to elevated local concentrations of free fatty acids, which can penetrate the coronary vessel wall and exacerbate lipid accumulation, smooth muscle cell proliferation, and foam cell formation [51]. Moreover, EAT has been implicated in promoting microvascular dysfunction through paracrine effects on coronary resistance vessels, potentially contributing to ischemia even in the absence of critical epicardial stenosis and amplifying the clinical impact of established atherosclerotic lesions [52].

The present meta-analysis adds an important methodological dimension to this pathophysiological framework by demonstrating that the association between EAT and obstructive CAD is robust regardless of whether EAT is measured in systole or diastole, while also suggesting that the magnitude of the association may vary according to the phase of the cardiac cycle. Cyclic changes in ventricular volume and myocardial wall tension influence the spatial configuration of epicardial fat, with systolic contraction compressing EAT against the myocardium and diastolic relaxation allowing relative expansion [53]. These mechanical dynamics may affect the apparent thickness of EAT and, potentially, the sensitivity of echocardiographic measurements in capturing biologically relevant fat burden.

From a mechanistic standpoint, systolic EAT measurements may preferentially reflect the component of epicardial fat that is tightly adherent to the coronary adventitia and myocardium, and thus more directly involved in paracrine signaling. Conversely, diastolic measurements may capture a larger apparent volume of epicardial fat influenced by ventricular relaxation and pericardial constraint. The finding that both systolic and diastolic EAT measurements are strongly associated with obstructive CAD supports the concept that overall epicardial fat burden—rather than a single static dimension—is pathophysiologically relevant. At the same time, the observed differences in effect size underscore that these measurements are not strictly interchangeable and may reflect partially distinct biological and mechanical aspects of EAT–coronary interactions.

Inflammatory activation within EAT is further modulated by traditional cardiovascular risk factors, including aging, male sex, insulin resistance, and visceral adiposity [54]. However, the absence of significant moderators in the meta-regression analysis suggests that the relationship between EAT thickness and obstructive CAD is relatively consistent across diverse demographic and metabolic profiles, reinforcing the notion that EAT represents an integrated marker of local coronary risk rather than a simple surrogate of systemic adiposity.

Collectively, these mechanistic considerations support a model in which EAT acts as a locally active, inflammation-driven amplifier of coronary atherosclerosis. Increased EAT thickness reflects not only excess visceral fat accumulation but also a pathogenic microenvironment capable of accelerating plaque development and progression. The robust association observed in this meta-analysis—across both systolic and diastolic measurements—reinforces the biological plausibility of EAT as a clinically relevant imaging biomarker of obstructive CAD, while highlighting the importance of methodological standardization to optimize its translational application.

### 4.3. Clinical Implications

The findings of the present meta-analysis have several relevant implications for clinical practice, particularly in the context of non-invasive risk stratification for obstructive CAD. The strong and consistent association between increased EAT thickness and angiographically confirmed obstructive CAD supports the integration of EAT assessment into routine TTE, especially in patients undergoing evaluation for suspected CAD. However, the very high between-study heterogeneity observed across pooled analyses indicates that EAT measurement should be interpreted with caution when applied to individual patients. Moreover, it should be acknowledged that the majority of included studies enrolled predominantly male patients referred to tertiary centers for invasive coronary angiography, thereby limiting the direct applicability of these findings to broader, lower-risk, or community-based populations.

From a practical standpoint, echocardiographic measurement of EAT represents an attractive imaging biomarker: it is inexpensive, widely available, reproducible, and can be obtained without additional imaging time, contrast agents, or radiation exposure. In patients with intermediate pre-test probability of CAD or equivocal clinical presentation, increased EAT thickness may provide incremental information beyond traditional risk factors and standard echocardiographic parameters, potentially prompting closer surveillance or more aggressive diagnostic strategies. In contrast, its role as a screening tool in asymptomatic individuals or in unselected general populations remains uncertain and is not directly supported by the current evidence base.

Although none of the studies included in the present meta-analysis systematically evaluated the association between EAT thickness and arrhythmic outcomes, emerging evidence suggests that increased EAT burden may be associated with adverse electrophysiological remodeling and increased arrhythmic risk, further supporting the potential clinical relevance of EAT assessment beyond purely anatomical coronary disease evaluation [55].

Importantly, the present analysis demonstrates that both systolic and diastolic EAT measurements are clinically informative, as each is significantly associated with obstructive CAD. This finding reassures clinicians that EAT assessment retains diagnostic value even in the absence of strict methodological uniformity. However, the observed differences in effect size between systolic and diastolic measurements indicate that these approaches are not fully interchangeable. Accordingly, systolic and diastolic EAT measurements should not be considered equivalent for clinical or research purposes, particularly when comparing results across studies, pooling data in meta-analyses, or proposing standardized acquisition protocols. Differences in measurement phase may introduce systematic variability that can influence effect estimates, diagnostic performance, and proposed cut-off values. As such, consistent reporting of the timing of EAT measurement within the cardiac cycle is essential for accurate interpretation, comparison across studies, and potential implementation of clinically meaningful cut-off values.

From a pragmatic clinical perspective, when technically feasible and image quality is adequate, diastolic EAT measurement may be preferred, given its numerically larger pooled effect size and potentially greater sensitivity in capturing epicardial fat expansion. However, systolic measurement remains a valid and clinically informative alternative, particularly in routine echocardiographic workflows where systolic frames are more consistently acquired and reproducible. Importantly, whichever phase is selected, consistent phase-specific measurement within the same laboratory and across serial examinations is likely more critical than the choice of phase itself.

The results also highlight the potential role of EAT as a marker of coronary atherosclerotic burden rather than a simple reflection of overall obesity. The observation that EAT thickness is strongly associated with CAD independently of BMI underscores its ability to capture a component of visceral adiposity that is more directly linked to coronary pathology [56]. This may be particularly relevant in patients with normal or mildly increased BMI but elevated cardiometabolic risk.

At present, EAT thickness should not be viewed as a standalone diagnostic tool for obstructive CAD. Rather, it should be interpreted as a complementary parameter, integrated with clinical assessment, laboratory findings, and conventional imaging results. Given the high heterogeneity of available evidence, EAT measurement is better suited for global risk stratification and phenotyping rather than for individual-level decision-making or threshold-based diagnosis. In this context, the lack of interchangeability between systolic and diastolic measurements further reinforces the need for phase-specific reference values and reporting standards.

Although standardized mean differences were used for statistical pooling to account for methodological heterogeneity, these effect sizes correspond, in practical terms, to absolute differences in EAT thickness of approximately 2–3 mm between CAD and non-CAD groups, as observed across most included studies. While such differences are easily detectable by echocardiography, they remain insufficiently precise to define universal screening thresholds or decision cut-offs at the individual patient level. Consequently, current evidence supports the use of EAT thickness as a relative risk marker rather than as a binary diagnostic parameter.

Finally, the present findings emphasize the need for greater methodological standardization before EAT measurement can be fully translated into clinical algorithms. Consensus regarding the optimal phase of measurement, anatomical landmarks, and reporting standards will be essential to ensure reproducibility and facilitate the adoption of EAT-based thresholds in clinical practice. Until such standardization is achieved, EAT thickness should be considered a valuable adjunctive marker that enhances, rather than replaces, established diagnostic pathways for CAD. Future studies should specifically evaluate the performance of EAT assessment in lower-risk populations, primary prevention settings, and female cohorts to better define its role beyond the angiography-referred population.

### 4.4. Future Directions

**Figure 6:**
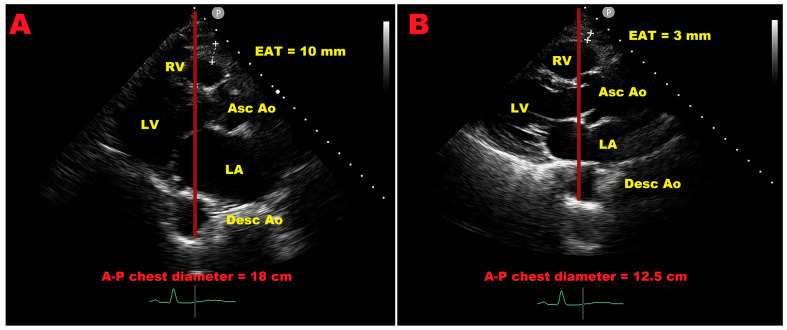
Influence of antero–posterior chest diameter on echocardiographic measurement of epicardial adipose tissue thickness. Representative transthoracic echocardiographic images illustrating the potential impact of thoracic geometry on systolic epicardial adipose tissue (EAT) thickness assessment. Panel (**A**) shows a subject with a wide antero–posterior (A–P) chest diameter (18 cm) and an increased apparent EAT thickness (10 mm), whereas panel (**B**) depicts a subject with a narrow A–P chest diameter (12.5 cm) and a markedly lower measured EAT thickness (3 mm), despite comparable imaging planes. These examples highlight how chest wall geometry may influence echocardiographic EAT measurements and should be considered when interpreting interindividual and interstudy variability.

An additional and potentially relevant research perspective concerns the interplay between chest wall conformation, EAT distribution, and echocardiographic measurement variability. As illustrated in Figure 6, thoracic geometry—particularly the antero–posterior (A–P) chest diameter—may influence the apparent thickness of systolic EAT measured by TTE. Subjects with a wider A–P chest diameter tend to exhibit greater measured EAT thickness, whereas individuals with a narrower thorax may show lower values despite comparable imaging planes. Such geometric factors may partly contribute to the substantial inter-study heterogeneity observed in the present meta-analysis and should be considered in future methodological standardization efforts.

Over the last several years, a modified Haller index (MHI) has been developed and validated as a non-radiological parameter for characterizing chest wall conformation using transthoracic echocardiography, showing good agreement with the conventional radiological Haller index [57] and applicability also in subjects with obesity [58]. Previous studies using this approach have demonstrated that thoracic geometry is associated with distinct cardiovascular phenotypes and risk profiles, with narrow A–P chest diameter generally linked to more favorable clinical characteristics and wider thoracic conformation associated with higher prevalence of adverse cardiovascular outcomes [59,60]. These observations support the hypothesis that chest wall morphology may influence epicardial fat distribution, with wider thoracic conformation potentially associated with greater EAT burden, independently of BMI. Consistent with this concept, body fat distribution patterns further modulate epicardial fat accumulation and myocardial mechanics, with android obesity being associated with increased visceral and epicardial adiposity compared with gynoid fat distribution [61].

Collectively, these findings suggest that integrating echocardiographic EAT assessment with thoracic geometry and anthropometric phenotype may improve the interpretability of EAT measurements and help reduce inter-individual and inter-study variability. Future prospective studies combining standardized EAT acquisition protocols with systematic assessment of chest conformation may further refine cut-off determination and enhance the translational value of epicardial fat assessment.

### 4.5. Methodological Limitations

Several methodological and technical limitations should be considered when interpreting the findings of this meta-analysis. First, the majority of included studies were single-center observational investigations, predominantly adopting prospective or cross-sectional designs, while only a minority were retrospective. Nevertheless, observational study designs are inherently susceptible to selection bias, residual confounding, and incomplete adjustment for relevant clinical covariates, which may limit the ability to infer a causal relationship between increased EAT thickness and obstructive CAD. In this context, the absence of randomized designs and standardized enrollment criteria may also have contributed to overestimation or underestimation of true effect sizes.

Second, although all studies used invasive coronary angiography as the reference standard for CAD diagnosis, the definition of obstructive disease was not entirely uniform, with stenosis thresholds ranging from ≥50% to ≥70% in at least one major epicardial coronary artery. This variability may have contributed to heterogeneity in effect estimates and limits the direct comparability of study populations. In addition, differences in patient referral patterns and clinical indications for angiography across centers may have influenced the prevalence and severity of CAD in the included cohorts. Such variability may have introduced spectrum bias, potentially affecting the magnitude of observed associations between EAT thickness and CAD.

From a technical perspective, echocardiographic assessment of EAT remains subject to several sources of variability. Although all studies used TTE, measurement protocols differed with respect to imaging planes, anatomical landmarks, and—most importantly—the timing of assessment within the cardiac cycle. While the present meta-analysis explicitly addressed systolic versus diastolic measurements, the lack of standardized acquisition protocols likely contributed to the substantial heterogeneity observed in both analyses. Furthermore, inter-vendor differences among echocardiographic platforms and software, as well as variability in image quality, frame rate, and caliper placement, may have affected measurement reproducibility [62]. Notably, only a minority of studies reported intra- or inter-observer variability, precluding a robust assessment of measurement reliability. Measurement imprecision may have introduced random error and contributed to dispersion of effect size estimates across studies.

Another important limitation relates to confounding. Several studies did not perform comprehensive multivariable adjustment for established cardiovascular risk factors, hemodynamic status, or markers of systemic inflammation. Although meta-regression analyses did not identify significant moderators among available study-level variables, the absence of individual patient data precluded more granular adjustment and interaction analyses. As such, unmeasured confounders—such as medication use, metabolic status, or inflammatory burden—may have influenced the observed associations. Importantly, incomplete confounder adjustment may not only affect the presence of an association but may also systematically bias the magnitude of pooled effect estimates.

The high degree of between-study heterogeneity represents an additional limitation. Although the direction of effect was consistent across all studies and results remained robust in sensitivity analyses, I^2^ values exceeded 90% in most pooled analyses, indicating substantial unexplained variability. This heterogeneity likely reflects a combination of methodological differences, population heterogeneity, and variability in EAT measurement techniques rather than true inconsistency of the association. Consequently, pooled standardized mean differences should be interpreted as summary measures of association rather than precise estimates of effect magnitude applicable across clinical settings.

Finally, despite the absence of statistically significant publication bias based on funnel plot inspection and Egger’s testing, the predominance of small observational studies raises the possibility that negative or neutral findings may be underrepresented in the literature. Consequently, the magnitude of the pooled effect sizes should be interpreted with caution.

Taken together, these limitations indicate that while the association between increased EAT thickness and obstructive CAD appears robust, the findings should be interpreted within the context of heterogeneous study designs and measurement protocols. In particular, the observational nature of all included studies and the presence of methodological shortcomings highlighted by the NIH Quality Assessment Tool suggest that pooled effect sizes may be influenced by residual bias and should not be directly translated into quantitative clinical decision thresholds. These considerations underscore the need for large, prospective, multicenter studies with standardized echocardiographic EAT assessment and comprehensive adjustment for confounding factors before EAT can be fully integrated into routine clinical risk stratification algorithms.

## 5. Conclusions

This systematic review and meta-analysis demonstrates a strong and consistent association between increased EAT thickness measured by TTE and angiographically confirmed obstructive CAD. Importantly, this relationship was observed for both systolic and diastolic EAT measurements, supporting epicardial fat accumulation as a robust imaging marker of coronary atherosclerotic burden at the population level.

By explicitly comparing EAT measurements obtained at different phases of the cardiac cycle, the present study addresses a relevant methodological gap in the existing literature. Although both systolic and diastolic assessments showed significant discriminatory capacity, differences in effect size suggest that these approaches are not fully interchangeable. This finding highlights the importance of measurement timing as a contributor to inter-study heterogeneity and underscores the need for standardized echocardiographic protocols.

From a clinical standpoint, EAT thickness represents a simple, non-invasive, and widely available parameter that may complement traditional cardiovascular risk factors and conventional imaging in the evaluation of patients with suspected CAD. However, given the observational design of the included studies and the substantial heterogeneity observed, EAT measurement should currently be considered an adjunctive marker rather than a standalone diagnostic tool. At present, evidence is insufficient to support routine implementation of EAT measurement as a screening or decision-making tool in the absence of validated cut-off values and standardized acquisition protocols.

Future large-scale, prospective studies with standardized acquisition methods, clearly defined timing within the cardiac cycle, and comprehensive adjustment for confounders are warranted to refine EAT-based thresholds and clarify its role in clinical risk stratification. In particular, future investigations should focus on reproducibility across centers and vendors, establishment of phase-specific normative ranges, and assessment of the incremental predictive value of EAT beyond established clinical risk scores and conventional imaging markers. Only through such dedicated validation efforts can EAT measurement be reliably translated into routine clinical practice.

## Figures and Tables

**Table 1:** Summary of included studies [21,22,23,24,25,26,27,28,29,30,31,32,33,34,35,36,37,38,39,40,41,42] comparing EAT thickness in CAD vs. non-CAD cohorts.

Study Name, Year and Country	Study Design	Size (n)	Mean Age (yrs)	Males (%)	Software	Timing EAT Assessment	EAT Cut-Off for Critical CAD (mm)
Jeong J.W. (2007), S. Korea [21]	Prospective, monocentric	CAD = 50 Ctrls = 153	CAD = 66.1 Ctrls = 62.2	CAD = 54 Ctrls = 52.9	GE	End-diastole	7.6
Ahn S.G. (2008), S. Korea [22]	Prospective, monocentric	CAD = 340 Ctrls = 267	CAD = 63 Ctrls = 54	CAD = 49 Ctrls = 52	Siemens	End-diastole	4
Eroglu S. (2009), Turkey [23]	Prospective, monocentric	CAD = 100 Ctrls = 50	CAD = 56.4 Ctrls = 54.1	CAD = 82 Ctrls = 18	Acuson	End-diastole	5.2
Mustelier J.V. (2011), Cuba [24]	Prospective, monocentric	CAD = 185 Ctrls = 65	CAD = 63 Ctrls = 58.7	CAD = 74.6 Ctrls = 40	Philips	End-systole	5.2
Yañez-Rivera T.G. (2014), Mexico [25]	Prospective, monocentric	CAD = 119 Ctrls = 34	CAD = 61.8 Ctrls = 59.2	CAD = 82.3 Ctrls = 41.1	Philips	End-systole	5.2
Wang T. (2014), China [26]	Prospective, monocentric	CAD = 149 Ctrls = 224	CAD = 69 Ctrls = 63	CAD = 83.9 Ctrls = 82.6	Philips	End-systole	4.7
Hirata Y. (2015), Japan [27]	Prospective, monocentric	CAD = 166 Ctrls = 145	CAD = 68 Ctrls = 66	CAD = 73 Ctrls = 60	GE	End-systole	4.2
Erkan A.F. (2016), Turkey [28]	Prospective, monocentric	CAD = 84 Ctrls = 51	CAD = 65 Ctrls = 51	CAD = 60 Ctrls = 33	GE	End-systole	5.75
Sinha S.K. (2016), India [29]	Prospective, monocentric	CAD = 464 Ctrls = 85	CAD = 60.3 Ctrls = 54.4	CAD = 63.3 Ctrls = NS	GE	End-systole	4.65
Meenakshi K. (2016), India [30]	Prospective, monocentric	CAD = 50 Ctrls = 50	CAD = 52.6 Ctrls = 51.5	CAD = 63 Ctrls = 64	Philips	End-systole	6.5
Ghaderi F. (2016), Iran [31]	Prospective, monocentric	CAD = 59 Ctrls = 41	CAD = 59.8 Ctrls = 57.2	CAD = 52.5 Ctrls = 61	GE	End-systole	4.25
Kim M.N. (2018), S. Korea [32]	Prospective, multicentric	CAD = 223 Ctrls = 328	CAD = 62.9 Ctrls = 58.2	CAD = 63.7 Ctrls = 41.6	GE	End-systole	NS
Tekin I. (2018), Turkey [33]	Prospective, monocentric	CAD = 48 Ctrls = 49	CAD = 61.6 Ctrls = 56.6	CAD = 63.3 Ctrls = 36.7	GE	End-diastole	5.5
Kamal D. (2018), Egypt [34]	Prospective, multicentric	CAD = 100 Ctrls = 50	CAD = 57.8 Ctrls = 44.8	CAD = 65 Ctrls = 42	GE	End-diastole	5.5
Verma B. (2019), India [35]	Prospective, monocentric	CAD = 250 Ctrls = 250	CAD = 55.2 Ctrls = 50.8	CAD = 84 Ctrls = 62.4	Acuson	End-systole and end-diastole	5 and 4
Turan Y. (2020), Turkey [36]	Prospective, monocentric	CAD = 109 Ctrls = 50	CAD = 59.6 Ctrls = 58.3	CAD = 59.6 Ctrls = 58	Philips	End-diastole	NS
Parisi V. (2020), Italy [37]	Prospective, monocentric	CAD = 340 Ctrls = 159	CAD = 63.9 Ctrls = NS	CAD = 56.9 Ctrls = NS	GE	End-systole	10
Shambu S.K. 2020), India [38]	Prospective, monocentric	CAD = 411 Ctrls = 92	CAD = 61.7 Ctrls = 57.4	CAD = 62.5 Ctrls = 67.4	Philips	End-systole	4.75
Mahmoud I. (2021), Germany [39]	Retrospective, monocentric	CAD = 196 Ctrls = 203	CAD = 62 Ctrls = 58	CAD = 46 Ctrls = 45	NS	End-systole	6.5
Jehn S. (2023), Germany [40]	Prospective, monocentric	CAD = 141 Ctrls = 516	CAD = 67 Ctrls = 55.6	CAD = 63.8 Ctrls = 50.2	Philips	End-systole	5.5
Braescu L. (2024), Romania [41]	Prospective, monocentric	CAD = 14 Ctrls = 11	CAD = 67.8 Ctrls = 61.8	CAD = 78.6 Ctrls = 63.6	GE	End-systole	7.25
Bouzidi H. (2025), Tunisia [42]	Prospective, monocentric	CAD = 80 Ctrls = 40	CAD = 60.9 Ctrls = NS	CAD = 71.7 Ctrls = NS	GE	End-systole and end-diastole	4.4 and 2.45

CAD = coronary artery disease; Ctrls, controls; GE, General Electric; EAT = epicardial adipose tissue; NS = not specified.

**Table 2:** Pooled demographic, clinical, laboratory, and echocardiographic characteristics of CAD and non-CAD cohorts included in the meta-analysis.

	Number of Studies for Parameter Assessed (Size)	CAD Group	Non-CAD Group	*p*-Value
*Demographics and anthropometrics*				
Age (years)	22 (3678 vs. 2913)	62.1 (52.6–69)	56.6 (44.8–66)	**<0.001**
Male sex (%)	22 (3678 vs. 2913)	66.4 (46–84)	51.1 (18–82.6)	**<0.001**
BMI (kg/m^2^)	18 (2645 vs. 2405)	27.4 (24–36)	26.6 (23.6–30.7)	0.27
WC (cm)	12 (1733 vs. 1483)	93.3 (82–103.5)	90.8 (78.9–101)	**<0.001**
WHR	4 (476 vs. 374)	0.96 (0.9–1.01)	0.93 (0.9–0.97)	**0.002**
*Cardiovascular risk factors*				
Hypertension (%)	19 (2993 vs. 2272)	63.1 (42.4–92.8)	47.5 (2–81.8)	**<0.001**
Diabetes (%)	19 (2794 vs. 2629)	38.8 (12–90)	21.3 (0–48)	**<0.001**
Dyslipidemia (%)	13 (1998 vs. 1885)	50.8 (17.9–78.6)	37.4 (0–90.9)	**<0.001**
Smoking (%)	19 (2794 vs. 2629)	41.6 (16–89.3)	29.3 (11.8–55.8)	**<0.001**
Family history of CAD (%)	7 (882 vs. 1304)	23.1 (7.4–29.1)	15.4 (0–33)	**0.001**
*Laboratory parameters*				
Hb (g/dL)	5 (471 vs. 593)	13.7 (13.3–14.5)	13.6 (12.9–14.4)	0.55
Creatinine (g/dL)	7 (873 vs. 1560)	1.03 (0.88–1.45)	0.86 (0.71–1.0)	**0.02**
FPG (mg/dL)	5 (578 vs. 330)	125.5 (103.2–147.6)	104.9 (96–118)	**0.001**
LDL-cholesterol (mg/dL)	14 (1989 vs. 2331)	118.3 (91.6–141.3)	116.4 (95.9–131.6)	0.42
HDL-cholesterol (mg/dL)	13 (1975 vs. 2320)	44.6 (35.8–55)	49.2 (42.2–61)	**<0.001**
Triglycerides (mg/dL)	14 (1989 vs. 2331)	168.5 (129–213.7)	150.3 (119–211.3)	**<0.001**
Uric acid (mg/dL)	4 (522 vs. 520)	6.3 (5.3–8.4)	5.2 (4.9–5.6)	**0.002**
HsCRP (mg/dL)	7 (1147 vs. 1201)	1.31 (0.26–3.0)	0.81 (0.2–2.35)	**0.003**
*Echocardiographic indices*				
LV mass (g)	3 (248 vs. 149)	100.4 (84.4–114.1)	95.7 (80.9–103.9)	0.19
LVEF (%)	7 (722 vs. 581)	52.8 (45–60)	58.4 (46.7–64.4)	**<0.001**
Systolic EAT (mm)	16 (2931 vs. 2294)	6.8 (5–12)	4.5 (3.1–8)	**<0.001**
Diastolic EAT (mm)	8 (1077 vs. 909)	5.7 (3.3–7.6)	3.5 (1.5–5.2)	**<0.001**
Systolic EAT cut-off (mm)	16 (2931)	5.6 (4.2–10)	/	/
Diastolic EAT cut-off (mm)	8 (1077)	4.9 (2.45–7.6)	/	/
*Clinical scores*				
Gensini score	6 (904 vs. 637)	44.6 (29.4–53.7)	10.9 (0–34.4)	**<0.001**
Syntax score	2 (334 vs. 301)	27.3 (25–29.7)	3.8 (0–7.7)	**<0.001**
*Current medical therapy*				
Antiplatelets (%)	6 (711 vs. 1180)	50.8 (19.9–78.6)	26.5 (12.6–47.8)	**<0.001**
BB (%)	6 (736 vs. 809)	40.6 (20–78.6)	33.6 (8.5–81.8)	**0.004**
CCB (%)	6 (728 vs. 1101)	40.6 (19–68.8)	27.9 (8–51.5)	**0.02**
ACE-i (%)	8 (1073 vs. 1528)	45.9 (30–68.8)	32.9 (14–57)	**0.007**
ARBs (%)	6 (728 vs. 1101)	40.4 (21.4–68.8)	28.1 (6–51.5)	**0.04**
Diuretics (%)	2 (114 vs. 61)	59.8 (34–85.7)	59.0 (18–100)	0.05
Nitrates (%)	2 (114 vs. 61)	32.9 (23–42.8)	5.5 (2–9.1)	**0.01**
Statins (%)	8 (1073 vs. 1528)	40.0 (12.1–85.7)	25.7 (11.3–45.5)	**<0.001**

Results are expressed as study-level medians with corresponding ranges. Statistically significant *p* values are shown in bold. ACE-i, angiotensin-converting enzyme inhibitors; ARBs, angiotensin receptor blockers; BB, beta-blockers; BMI, body mass index; CAD, coronary artery disease; CCB, calcium channel blockers; EAT, epicardial adipose tissue; FPG, fasting plasma glucose; Hb, hemoglobin; HDL, high-density lipoprotein; HsCRP, high-sensitivity C-reactive protein; LDL, low-density lipoprotein; LV, left ventricular; LVEF, left ventricular ejection fraction; WC, waist circumference; WHR, waist-to-hip ratio.

**Table 3:** Meta-regression analysis of covariates influencing effect size estimates.

Covariate	Coefficient	Standard Error	95% Lower	95% Upper	*p*-Value
Intercept	−2.539	8.922	−20.025	14.947	0.776
Age	0.005	0.066	−0.125	0.135	0.943
Male sex	−0.020	0.022	−0.063	0.023	0.356
Hypertension	0.015	0.026	−0.036	0.066	0.566
Diabetes	0.016	0.016	−0.015	0.047	0.319
Smoking	−0.017	0.020	−0.055	0.022	0.402
LDL-cholesterol	0.031	0.024	−0.016	0.077	0.194
Triglycerides	0.256	0.432	−0.590	1.102	0.553
WC	−0.008	0.015	−0.038	0.022	0.597
Asian Country	0.017	0.066	−0.113	0.147	0.798
Systolic timing	0.133	0.608	−1.059	1.325	0.827
Non-GE software	0.009	0.474	−0.920	0.938	0.985
Cut-off stenosis ≥50%	0.276	0.394	−0.497	1.048	0.484

GE, General Electric; LDL, low-density lipoprotein; WC, waist circumference.

## Data Availability

Data extracted from included studies will be publicly available on Zenodo (https://zenodo.org).

## Supplementary Materials

The following supporting information can be downloaded at https://www.mdpi.com/article/10.3390/jcm15020878/s1, Supplementary Materials S1: PRISMA 2020 checklist; Supplementary Materials S2: The full INPLASY record (https://inplasy.com/inplasy-2026-1-0009/, accessed on 3 January 2026); Supplementary Materials S3: NIH Quality Assessment of included studies [21,22,23,24,25,26,27,28,29,30,31,32,33,34,35,36,37,38,39,40,41,42].
