# Supplementary material for: Systolic Versus Diastolic Echocardiographic Assessment of Epicardial Adipose Tissue for the Detection of Obstructive Coronary Artery Disease: A Systematic Review and Meta-Analysis
Source: J Clin Med. 2026 Jan 21;15(2):878. doi: 10.3390/jcm15020878 (PMC12841758; doi:10.3390/jcm15020878)
Supplement: Supplementary file 1 [file jcm-15-00878-s001.zip › Supplementary Materials S2.pdf]

INPLASY

# Systolic versus Diastolic Echocardiographic Assessment of Epicardial Adipose Tissue for the Detection of Obstructive Coronary Artery Disease: A Systematic Review and Meta-Analysis

INPLASY202610009
doi: 10.37766/inplasy2026.1.0009
Received: 3 January 2026
Published: 3 January 2026

Sonaglioni, A; Gramaglia, G; Nicolosi, GL; Lombardo, M; Baravelli, M.

**Corresponding author:**
Andrea Sonaglioni

sonaglioniandrea@gmail.com

**Author Affiliation:**
MultiMedica.

**ADMINISTRATIVE INFORMATION**

**Support -** Ministero della Salute.

**Review Stage at time of this submission -** Preliminary searches.

**Conflicts of interest -** None declared.

**INPLASY registration number:** INPLASY202610009

**Amendments -** This protocol was registered with the International Platform of Registered Systematic Review and Meta-Analysis Protocols (INPLASY) on 3 January 2026 and was last updated on 3 January 2026.

## INTRODUCTION

**Review question / Objective** This systematic review and meta-analysis has been designed to evaluate the association between echocardiographically measured EAT thickness and angiographically confirmed obstructive CAD, with specific focus on systolic versus diastolic assessments.

**Rationale** Previous meta-analyses have primarily examined the relationship between EAT and CAD without accounting for the timing of echocardiographic assessment, implicitly assuming equivalence between systolic and diastolic measurements. This assumption may partly explain the substantial heterogeneity reported across studies and limits the clinical interpretability of pooled estimates. A systematic comparison of systolic versus diastolic EAT measurements is therefore needed to determine whether these approaches capture the same biological signal or reflect distinct aspects of epicardial fat–coronary interactions. Accordingly, the present systematic review and meta-analysis aimed to evaluate the association between echocardiographically measured epicardial adipose tissue thickness and angiographically confirmed obstructive CAD, with a specific focus on comparing measurements obtained during systole and diastole. By conducting separate quantitative syntheses based on the timing of EAT assessment within the cardiac cycle, we aim to clarify whether systolic and diastolic EAT measurements differ in their ability to discriminate patients with obstructive CAD from those without significant coronary stenosis. We hypothesized that while both measurements would be significantly associated with obstructive CAD, the strength of this association would vary according to the phase of the cardiac cycle in which EAT is measured.

**Condition being studied** Obstructive coronary artery disease (CAD) remains a leading cause of morbidity and mortality worldwide, despite substantial advances in preventive strategies, diagnostic imaging, and revascularization

Sonaglioni et al. INPLASY protocol 202610009. doi:10.37766/inplasy2026.1.0009 Downloaded from https://inplasy.com/inplasy-2026-1-0009/

techniques. Early identification of individuals at increased risk of obstructive CAD is therefore of paramount importance, particularly in clinical contexts where traditional cardiovascular risk factors alone may not fully explain individual susceptibility to coronary atherosclerosis.

Epicardial adipose tissue (EAT) has gained increasing attention as a metabolically active visceral fat depot located between the myocardium and the visceral layer of the pericardium, surrounding the epicardial coronary arteries. Unlike other adipose compartments, EAT shares a common microcirculation with the underlying myocardium and lacks a separating fascial plane, allowing direct paracrine and vasocrine interactions with the coronary vasculature. Through the secretion of pro-inflammatory cytokines, adipokines, and free fatty acids, EAT has been implicated in endothelial dysfunction, plaque formation, and progression of coronary atherosclerosis. Accordingly, increased EAT thickness has been consistently associated with both the presence and angiographic severity of CAD across multiple imaging modalities.

Transthoracic echocardiography represents the most widely available, cost-effective, and clinically applicable technique for EAT assessment. Nevertheless, despite its broad adoption, considerable heterogeneity persists in echocardiographic EAT quantification, particularly regarding the timing of measurement within the cardiac cycle. Some investigators measure EAT thickness at end-systole, when epicardial fat is maximally compressed against the myocardium, whereas others perform measurements at end-diastole, when ventricular relaxation alters the spatial relationship between EAT, myocardium, and pericardium. These approaches are often considered interchangeable, although they reflect different mechanical and anatomical conditions.

Importantly, the behavior of epicardial adipose tissue is not static throughout the cardiac cycle. Cyclic changes in ventricular volume, myocardial wall stress, and pericardial constraint may influence the apparent thickness of EAT, with potential implications for measurement reproducibility and its association with coronary pathology. Whether systolic and diastolic EAT measurements convey equivalent information regarding obstructive CAD risk therefore remains uncertain. Although individual studies have demonstrated significant associations between EAT thickness and CAD using either systolic or diastolic measurements, no consensus has emerged as to which phase of the cardiac cycle provides superior discriminatory or predictive value.

## METHODS

**Search strategy** A comprehensive literature search will be independently performed by two investigators (A.S. and G.G.) to identify all relevant studies evaluating epicardial adipose tissue thickness in patients with and without obstructive coronary artery disease. PubMed, Embase, and Scopus databases will be systematically searched from inception to December 2025.

The search strategy will combine controlled vocabulary and free-text terms related to epicardial fat, coronary artery disease, and echocardiography. The following keywords and Boolean operators will be used: “epicardial adipose tissue” OR “epicardial fat” OR “epicardial fat thickness” AND “coronary artery disease” OR “obstructive coronary artery disease” OR “coronary stenosis” OR “coronary angiography” AND “echocardiography” OR “transthoracic echocardiography.”

No restrictions will be applied regarding language, publication date, or study design. To ensure completeness, the reference lists of all eligible articles and relevant review papers will be manually screened for additional studies not captured by the initial database search.

Any disagreements between reviewers during the study selection process will be resolved through consensus. When consensus could not be reached, a third investigator will be consulted to adjudicate eligibility.

**Participant or population** CAD cohorts vs. non-CAD cohorts.

**Intervention** Systolic versus Diastolic Echocardiographic Assessment of Epicardial Adipose Tissue in CAD vs. non-CAD cohorts.

**Comparator** Non-CAD cohorts.

**Study designs to be included** Case-control studies.

**Eligibility criteria** Studies will be considered eligible for inclusion if they will meet all of the following criteria:

(1) observational study design (case–control or cross-sectional) directly comparing patients with angiographically confirmed obstructive coronary artery disease (CAD) and individuals without significant coronary stenosis; (2) quantitative assessment of epicardial adipose tissue (EAT) thickness performed by transthoracic echocardiography;

(3) diagnosis of obstructive CAD established by invasive coronary angiography, using predefined

Sonaglioni et al. INPLASY protocol 202610009. doi:10.37766/inplasy2026.1.0009 Downloaded from https://inplasy.com/inplasy-2026-1-0009/

thresholds of luminal stenosis in at least one major epicardial coronary artery; and (4) availability of extractable quantitative data on EAT thickness for both CAD and non-CAD groups, reported either as mean ± standard deviation or in a format allowing data transformation.

Studies will be excluded if they will meet any of the following criteria: (1) assessment of EAT using imaging modalities other than transthoracic echocardiography, such as cardiac computed tomography or cardiac magnetic resonance imaging, without echocardiographic measurements; (2) inclusion of heterogeneous populations without a clearly defined comparison between obstructive CAD and non-obstructive or normal coronary arteries; (3) absence of invasive coronary angiography as the reference standard for CAD diagnosis; (4) lack of quantitative EAT measurements or insufficient data to calculate effect estimates; and (5) non-original publications, including conference abstracts, editorials, letters, case reports, or narrative reviews.

**Information sources** PubMed, Embase, and Scopus databases.

**Main outcome(s)** By conducting separate quantitative syntheses based on the timing of EAT assessment within the cardiac cycle, we aim to clarify whether systolic and diastolic EAT measurements differ in their ability to discriminate patients with obstructive CAD from those without significant coronary stenosis.

**Data management** Two reviewers (A.S. and G.G.) will independently screen all retrieved records by title and abstract, followed by a full-text evaluation of potentially eligible articles. Study selection will be performed according to the predefined eligibility criteria, and any disagreement will be resolved through discussion and consensus.

Data extraction will be carried out independently by the same reviewers using a standardized and predefined data collection form. Extracted information will include: (1) study characteristics, such as first author, year of publication, country, study design, and sample size of the CAD and non-CAD groups; (2) demographic and anthropometric variables, including age, sex distribution, body mass index, waist circumference, and waist-to-hip ratio, when available; (3) cardiovascular risk factors, namely hypertension, diabetes mellitus, dyslipidemia, active smoking, and family history of coronary artery disease; (4) laboratory parameters, including hemoglobin, serum creatinine, fasting plasma glucose, lipid profile components (LDL cholesterol, HDL cholesterol, triglycerides), uric acid, and high-sensitivity C-reactive protein; (5) echocardiographic parameters, comprising left ventricular ejection fraction, left ventricular mass, and epicardial adipose tissue thickness assessed by transthoracic echocardiography, with systolic and diastolic EAT measurements extracted separately when reported, together with any study-specific EAT cut-off values associated with obstructive or severe coronary artery disease; (6) clinical and angiographic indices, such as Gensini and SYNTAX scores, reflecting coronary atherosclerotic burden and lesion complexity; (7) baseline medical therapy, including use of antiplatelet agents, beta-blockers, calcium channel blockers, angiotensin-converting enzyme inhibitors, angiotensin receptor blockers, diuretics, nitrates, and statins; and (8) summary statistics, including mean ± standard deviation or median with interquartile range, along with corresponding p-values or confidence intervals.

All extracted data will be cross-checked for accuracy, and discrepancies between reviewers will be resolved by re-examination of the original articles until consensus will be achieved.

**Quality assessment / Risk of bias analysis** The methodological quality and risk of bias of the included studies will be independently assessed by two investigators (A.S. and G.L.N.) using the National Institutes of Health (NIH) Quality Assessment Tool for Case–Control Studies. This instrument evaluates multiple domains related to study design, population selection, exposure assessment, and control of confounding.

Based on the number of criteria fulfilled, each study will be assigned an overall quality rating of “good,” “fair,” or “poor” in accordance with NIH recommendations. Inter-rater agreement between reviewers will be quantified using Cohen’s kappa coefficient (κ). Any disagreements in individual domain judgments or overall quality classification will be resolved through discussion until consensus will be reached.

**Strategy of data synthesis** For descriptive purposes, continuous variables will be summarized as medians with interquartile ranges (IQRs), while categorical variables will be reported as counts and percentages. Comparative meta-analyses will be performed using random-effects models. For continuous variables, pooled estimates will be expressed as standardized mean differences (SMDs), whereas odds ratios will be calculated for categorical variables when applicable. Statistical significance of pooled effects will be assessed using Z-tests.

Sonaglioni et al. INPLASY protocol 202610009. doi:10.37766/inplasy2026.1.0009 Downloaded from https://inplasy.com/inplasy-2026-1-0009/

**Subgroup analysis** Separate meta-analyses will be conducted according to the timing of EAT measurement within the cardiac cycle (systolic versus diastolic assessment). A random-effects model based on the DerSimonian–Laird method will be selected a priori to account for anticipated clinical and methodological heterogeneity among studies.

**Sensitivity analysis** Sensitivity analyses will be conducted using a leave-one-out approach, sequentially excluding individual studies to assess the stability and robustness of the pooled estimates.

**Language restriction** No language restriction.

**Country(ies) involved** Italy.

**Keywords** epicardial adipose tissue; coronary artery disease; echocardiography; obstructive coronary stenosis; meta-analysis.

**Contributions of each author**

Author 1 - Andrea Sonaglioni - Author 1 drafted the manuscript.
Email: sonaglioniandrea@gmail.com
Author 2 - Giulio Gramaglia - The author contributed to the Search Strategy.
Email: giulio.gramaglia@unimi.it
Author 3 - Gian Luigi Nicolosi - The Author revised the original manuscript.
Email: gianluigi.nicolosi@gmail.com
Author 4 - Michele Lombardo - The author read, provided feedback and approved the final manuscript.
Email: michele.lombardo@multimedica.it
Author 5 - Massimo Baravelli - The author read, provided feedback and approved the final manuscript.
Email: massimo.baravelli@multimedica.it

Sonaglioni et al. INPLASY protocol 202610009. doi:10.37766/inplasy2026.1.0009 Downloaded from https://inplasy.com/inplasy-2026-1-0009/
