# Supplementary material for: Systolic Versus Diastolic Echocardiographic Assessment of Epicardial Adipose Tissue for the Detection of Obstructive Coronary Artery Disease: A Systematic Review and Meta-Analysis
Source: J Clin Med. 2026 Jan 21;15(2):878. doi: 10.3390/jcm15020878 (PMC12841758; doi:10.3390/jcm15020878)
Supplement: Supplementary file 1 [file jcm-15-00878-s001.zip › Supplementary Materials S3.pdf]

Study Name	Q1	Q2	Q3	Q4	Q5	Q6	Q7	Q8	Q9	Q10	Q11	Q12	Quality score
**Jeong J.W. (2007)**	YES	YES	NO	YES	YES	YES	NA	YES	NO	YES	YES	YES	**9 (Good)**
**Ahn S.G. (2008)**	YES	YES	NO	YES	YES	YES	NA	YES	NO	YES	NR	YES	**8 (Fair)**
**Eroglu S. (2009)**	YES	YES	NO	YES	YES	YES	NA	YES	NO	YES	YES	YES	**9 (Good)**
**Mustelier J.V. (2011)**	YES	YES	NO	YES	YES	YES	NR	YES	NO	YES	YES	YES	**9 (Good)**
**Yañez-Rivera T.G. (2014)**	YES	YES	NO	YES	YES	YES	NR	YES	NO	YES	YES	YES	**9 (Good)**
**Wang T. (2014)**	YES	YES	NO	YES	YES	YES	NR	YES	NO	YES	YES	YES	**9 (Good)**
**Hirata Y. (2015)**	YES	YES	NO	YES	YES	YES	NA	YES	NO	YES	YES	YES	**9 (Good)**
**Erkan A.F. (2016)**	YES	YES	NO	YES	YES	YES	NA	YES	NO	YES	YES	YES	**9 (Good)**
**Sinha S.K. (2016)**	YES	YES	NO	YES	YES	YES	NA	YES	NO	YES	NR	YES	**8 (Fair)**
**Meenakshi K. (2016)**	YES	YES	NO	YES	YES	YES	NA	YES	NO	YES	YES	YES	**9 (Good)**
**Ghaderi F. (2016)**	YES	YES	YES	YES	YES	YES	NR	YES	NO	YES	NR	YES	**9 (Good)**
**Kim M.N. (2018)**	YES	YES	NO	YES	YES	YES	NA	YES	NO	YES	YES	YES	**9 (Good)**
**Tekin I. (2018)**	YES	YES	NO	YES	YES	YES	NA	YES	NO	YES	YES	YES	**9 (Good)**
**Kamal D. (2018)**	YES	YES	NO	YES	YES	YES	NA	YES	NO	YES	YES	YES	**9 (Good)**
**Verma B. (2019)**	YES	YES	NO	YES	YES	YES	NA	YES	NO	YES	YES	YES	**9 (Good)**
**Turan Y. (2020)**	YES	YES	NO	YES	YES	YES	NA	YES	NO	YES	NR	YES	**8 (Fair)**
**Parisi V. (2020)**	YES	YES	YES	YES	YES	YES	NA	YES	NO	YES	YES	YES	**10 (Good)**
**Shambu S.K. (2020)**	YES	YES	NO	YES	YES	YES	NR	YES	NO	YES	YES	YES	**9 (Good)**
**Mahmoud I. (2021)**	YES	YES	YES	YES	YES	YES	NA	YES	NO	YES	YES	YES	**10 (Good)**
**Jehn S. (2023)**	YES	YES	YES	YES	YES	YES	NA	YES	NO	YES	YES	YES	**10 (Good)**
**Braescu L. (2024)**	YES	YES	NO	YES	YES	YES	NA	YES	NO	YES	YES	YES	**9 (Good)**
**Bouzidi H. (2025)**	YES	YES	NO	YES	YES	YES	NA	YES	NO	YES	YES	YES	**9 (Good)**
